# Retrograde Intramedullary Kirschner Wire Fixation as an Alternative for Treating Distal Fibular Shaft Fractures Combined with Distal Tibial Pilon Fractures

**DOI:** 10.3390/jpm12071124

**Published:** 2022-07-10

**Authors:** Cheng-Wei Huang, Wen-Tien Wu, Tsai-Chiu Yu, Ing-Ho Chen, Jen-Hung Wang, Kuang-Ting Yeh

**Affiliations:** 1 Department of Orthopedics, Hualien Tzu Chi Hospital, Buddhist Tzu Chi Medical Foundation, Hualien 970473, Taiwan; yelweche@gmail.com (C.-W.H.); timwu@tzuchi.com.tw (W.-T.W.); feyu@tzuchi.com.tw (T.-C.Y.); ihchen@tzuchi.com.tw (I.-H.C.); 2 School of Medicine, Tzu Chi University, Hualien 970374, Taiwan; 3 Department of Medical Research, Hualien Tzu Chi Hospital, Buddhist Tzu Chi Medical Foundation, Hualien 970473, Taiwan; paulwang@tzuchi.com.tw

**Keywords:** distal fibular fracture, pilon fracture, retrograde intramedullary pinning, lateral distal tibial angle (LDTA), anterior distal tibial angle (ADTA)

## Abstract

Treatment or management techniques for pilon fractures are associated with high complication rates and poor outcomes. No consensus exists regarding the optimal surgical option for pilon fractures, especially for pilon fractures combined with distal fibular fractures. Accordingly, we explored the use of fibular fixation for treating pilon fractures involving distal fibular shaft fractures. We hypothesized that retrograde intramedullary Kirschner wire (K-wire) fixation is a suitable alternative technique for distal fibular fixation. We retrospectively reviewed the data of 156 patients who underwent surgery for pilon fractures at our hospital from May 2013 to May 2021. The radiographic and functional outcomes were comparable between the fibular intramedullary nailing (Group A; *n* = 80) and the fibular plating (Group B; *n* = 76) groups. Groups A and B differed significantly in total hospitalization time (11.4 vs. 18.2 days, *p* = 0.024), length of postoperative admission (6.8 vs. 11.4 days, *p* = 0.012), and total admission cost (USD 3624 vs. USD 6145, *p* = 0.004). We also noted that poor Olerud and Molander ankle scores were significantly associated with age (*p* = 0.008), smoking (*p* = 0.012), and preoperative admission length (*p* = 0.018). Retrograde intramedullary K-wire fixation produced a comparable 12-month functional outcome to plate fixation for distal fibular shaft fractures, rendering it a viable alternative method based on soft tissue condition.

## 1. Introduction

Pilon fractures [1,2], also known as tibial plafond fractures, account for 7% of all tibial fractures [3]. Pilon fractures result from high-energy axial loading or low-energy rotational force, and 66% of such fractures also involve concomitant distal fibular fractures [4]. Despite advances in treatment techniques and devices, treating these fractures remains a challenge [5]. These fractures are also associated with high complication rates and poor outcomes, which can be attributed to articular comminution, metaphyseal osseous deficits, and circumferential soft tissue compromise.

Vital treatment goals for pilon fractures include anatomic articular restoration, mechanical axis restoration, adequate stabilization, and soft tissue management [6]. However, balancing these goals is difficult. A high-profile plating system may provide adequate stability but increase the risk of soft tissue complications. Studies have attempted to determine the optimal timing of surgical intervention in an attempt to improve outcomes [7,8,9], but their results remain controversial.

Intramedullary Kirschner wire (K-wire) fixation has been extensively used to treat simple distal fibular fractures. Nevertheless, research into the application of this method for treating combined pilon fractures is scant. This low-profile fixation method can maintain the length of the fibula without violating circumferential soft tissue. It may also provide adequate stability after postoperative nonweight-bearing rehabilitation. Accordingly, we investigated the use of K-wire fixation for treating pilon fractures involving distal fibular shaft fractures. We hypothesized that the use of intramedullary K-wires for fibular fixation could provide stable tibial fixation and, thus, afford favorable functional and radiographic outcomes while having relatively low complication rates.

## 2. Materials and Methods

**Figure 1:**
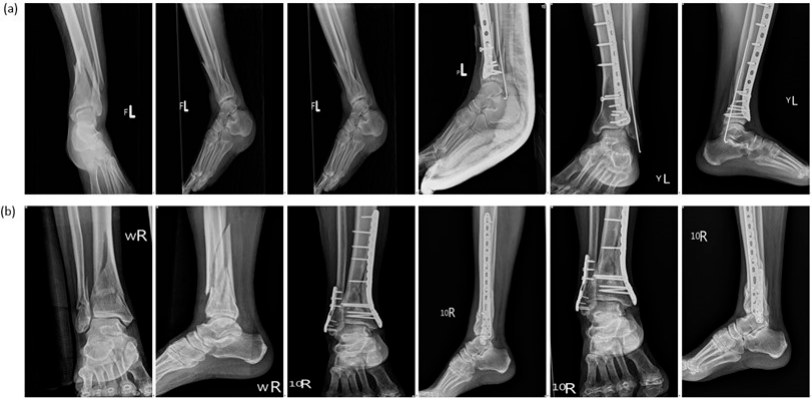
Patients were classified into two groups: (**a**) Group A—pilon fracture was fixed with the locking plate, and the distal fibula fracture was fixed with retrograde 2.0 mm K-pins; (**b**) Group B—pilon fractures and distal fibula fractures were both fixed with the locking plates.

We retrospectively reviewed patients who underwent surgery for both pilon fractures and distal fibular fractures at our institution between May 2013 and May 2021. The inclusion criteria were as follows: being musculoskeletally mature patients sustaining both pilon fractures (AO/OTA 43A to 43C) [10] and distal fibular shaft fractures (Danis–Weber type B or C) [11], as diagnosed through computed tomography (CT). Pilon fractures were treated using locking plates, regardless of type or brand, to achieve definitive fixations. By contrast, distal fibular shaft fractures were treated using either one retrograde intramedullary K-wire or a locking plate (Figure 1).

The method of retrogradely intramedullary K-pin for distal fibular fracture was applied in two situations: (1). The fibular fractures could be reduced well closely or through a stabbed wound (smaller than 2 cm). (2). The fracture site of the fibular was between the distal tibiofibular joint and the equal level of the most proximal end of the distal tibia fracture site. We checked the rotational alignment and ankle valgus deformity with preoperative computed tomography and preoperative and intraoperative X-ray of anteroposterior and lateral view. The uninjured contralateral ankle was referenced as the length reconstruction of the fractured fibula. For the K-pin length that we used in this study, it primarily depended on the patients’ fibula length and the extension of the fracture line. In general, the K-pin would be extended from the junction of the distal third to the middle third junction of the fibula. In our experience, a 2.0 mm K-pin will be the most suitable device for intramedullary pinning. Sometimes we will change to 1.8 mm or 2.4 mm K-pins under the feeling of tightness when passing the junction of the distal third to the middle third of the fibular shaft. All patients received at least 3 months of aggressive rehabilitation under no weight-bearing and skin condition monitoring and, subsequently, at least 3 months of partial weight-bearing under ankle brace protection. The K-pin will be removed at least 6 months after surgery if irritation or maybe earlier than 6 months if wound infection occurs.

Patients who were treated using external fixators, conventional plates, or tibial intramedullary nails as the final fixation devices were excluded. Moreover, patients who had insufficient data or radiography records, had a follow-up interval of <3 months, or required revision surgery were excluded, as were those who had prolonged hospital stays caused by other traumatic injuries or pre-existing medical conditions. Gustilo–Anderson type III open fractures may require prolonged soft tissue or neurovascular management [12]; therefore, we also excluded patients with these.

After applying our exclusion criteria, we enrolled 88 patients in the study. The patients’ medical records were reviewed to obtain data on their age, sex, past medical history, injury mechanism (high or low), and days of hospital stay, including the preoperative admission period and the postoperative admission period. Open fractures were marked and classified on the basis of the Gustilo–Anderson classification system.

**Figure 2:**
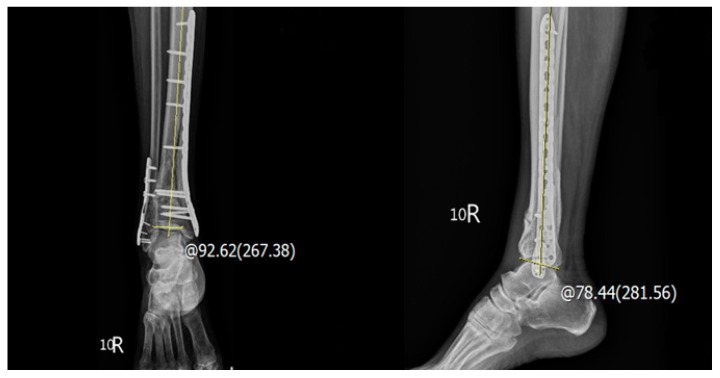
The postoperative angle measurement: LDTA, the distal tibial articular surface and the anatomical axis of the tibia in the coronal plane (normal values 89° ± 3°); ADTA, the mechanical axis of the tibia and the joint orientation line of the ankle in the sagittal plane (normal values 80° ± 3°).

Imaging analysis was conducted on both ankle and leg radiographs and CT scans. Our preoperative evaluation included classifying the pilon fractures on the basis of the AO/OTA classification system and determining the types of fibular fractures (comminuted, oblique, or transverse) [13]. The types of fixation devices on the tibia (medial or lateral plates) and fibula (locking plates or K-wires) were documented by assessing postoperative radiographs. Using radiographs acquired at the 12-month follow-up time point, we conducted lateral distal tibial angle (LDTA) and anterior distal tibial angle (ADTA) measurements. The LDTA was measured by determining the angle produced by the intersection of the line from the central axis of the tibia and a second line drawn across the epiphyseal surface of the distal tibia in the sagittal plane, while the ADTA was measured by determining the angle produced by the intersection of the line from the central axis of the tibia and a second line drawn across the epiphyseal surface of the distal tibia in the coronal plane, respectively (Figure 2) [14]. If the LDTA or ADTA differed from the normal angle (89° and 80°) by >10°, we determined the existence of an incongruent joint. All measurements were performed and confirmed by all authors.

We also considered common complications such as delayed union, post-traumatic osteoarthritis, and wound complications separately. Delayed union was defined as the absence of radiographic progression of healing 3 months after injury [15]. Post-traumatic ankle osteoarthritis was defined as joint space narrowing, osteophyte presence, and subchondral bone sclerosis, along with symptoms of persistent pain, swelling, or limited range of motion on the patient’s latest radiograph [16]. Any wound complications, including delayed healing, wound dehiscence, superficial wound infection, or osteomyelitis, were included in our study.

For the initial postoperative outcome assessment, we quantified the frequency of the patients’ outpatient clinic follow-ups that included analgesic prescriptions. A high frequency was defined as at least six visits in the first 6 months (regular outpatient visits). The patients were administered the Olerud and Molander Ankle Score (OMAS) questionnaire [17] at the 12-month outpatient follow-up to record self-reported outcomes.

Statistical analyses were performed using SPSS for Windows, version 23.0 (IBM Corp., Armonk, NY, USA). Descriptive statistics (means, standard deviations, ranges, coefficients of variation, and proportions) were used to describe the data. Moreover, an independent *t*-test was used for comparative analyses. Linear regression and the Pearson chi-square test were used for correlation analyses.

## 3. Results

We included 156 patients, including 94 males and 62 females, and divided them into two groups, namely Groups A (*n* = 80) and B (*n* = 76). Group A comprised patients who underwent distal fibular shaft fixation using retrograde intramedullary K-wires, and Group B comprised patients who underwent distal fibular shaft fixation with the locking plates. The mean (range) ages of the patients in Groups A and B were 54.1 (17–81) and 55.4 (16–87) years, respectively. A total of 38 of the patients were older than 65 years old, and 17 of them had a smoking habit. There were 9 patients with chronic renal failure, 14 patients with DM, and 44 patients with hypertension. There was no significance between the aforementioned demographic data of both groups (Table 1).

In total, 65.0% of the patients in Group A and 71.1% of those in Group B were caused by high injury mechanisms, respectively. Group A had a slightly higher proportion of open fractures than did Group B (20.0% vs. 17.1%), but the difference was not significant. In addition, the proportions of comminuted fibular fractures were similar between Groups A and B (32.5% vs. 36.8%). Regarding the AO/OTA classification of pilon fractures, Group A had fewer patients with type C fractures compared with Group B (22.5% vs. 27.7%); nevertheless, the difference was nonsignificant (Table 2).

The average total hospitalization time in Group A was 11.4 days, whereas that in Group B was 18.2 days, indicating a significant difference (*p* = 0.024), while the total admission costs were on average USD 3624 in Group A and USD 6145 in Group B, indicating a significant difference (*p* = 0.004). Furthermore, the average time of postoperative stay was in Group A 6.8 days, whereas that in Group B was 11.4 days, also indicating a significant difference (*p* = 0.012). The hospitalization time before operation was slightly longer in Group B (5.6 days) than in Group A (4.8 days), but this difference was nonsignificant (*p* = 0.263; Table 3). Postoperative complications were similar between the groups. The rate of delayed union was slightly higher in Group B (18.4%) than in Group A (13.8%), but the rate of post-traumatic osteoarthritis was higher in Group A than in Group B (37.5% to 32.9%). In addition, Group B had a higher rate of wound complications than did Group A (32.9% vs. 22.5%), but the difference was not statistically significant. Notably, fibular wound complications occurred only in Group B, with five patients experiencing fibular site infections. Regarding radiographic outcomes, the proportion of patients with joint incongruence in the sagittal plane was 15.0% in Group A and 13.2% in Group B, whereas that of those with joint incongruence in the coronal plane was 17.5% in Group A and 17.1% in Group B (Table 3), indicating a nonsignificant difference. Regarding postoperative outcomes, both groups revealed favorable results. Only 17.5% of the patients in Group A and 15.8% of those in Group B had a high frequency of outpatient visits, respectively, indicating a nonsignificant difference. The average OMAS values in Groups A and B were 75.4 and 77.2, respectively, also indicating a nonsignificant difference (Table 3). Tolerable K-pin irritated feelings were noted in 13 patients (16.2%) in Group A, but additional arrangement of surgical removal was not necessary for all patients.

Concerning factors associated with the postoperative functional outcome, age (*p* = 0.008), time before operation (preoperative admission period) (*p* = 0.018), and smoking (*p* = 0.012) exhibited a significant association with poor OMAS scores based on the multivariate logistical regression analysis. The different fibular fixation techniques were not associated with poor OMAS scores (Table 4).

## 4. Discussion

Pilon fracture management is among the most challenging problems faced by orthopedic traumatologists. Pilon fracture treatment strategies could have complication rates as high as 50% [18]. Despite the evolution of implants and treatment techniques, surgical outcomes have remained poor, and even when treatment is successfully completed, persistent dysfunction [19], poor health-related quality of life [20], and significant socioeconomic burden [21] may manifest.

De las Heras-Romero et al. emphasized reduction quality as the only modifiable factor contributing to successful treatment outcomes [22]. However, debate still persists regarding the best surgical strategies. Treatment techniques, including delayed single-stage surgery, external fixation alone, external fixation with limited articular reduction and fixation, and two-stage reconstruction, have all demonstrated success but still had some complications.

The necessity of fibular fixation is also debatable. Ruedi and Allgower stressed the importance of analyzing fibular fracture types to determine the injury mechanism before deciding on suitable surgical options [23]. Nonetheless, Kurylo et al. recently reported fibular fixation to be unnecessary in nonrotational pilon fractures [24]. Strauss et al. designed a cadaveric biomechanical examination, which revealed that an intact fibula might improve fixation stability in distal tibial fractures [25]. In the present study, we determined that fibular fixation played a pivotal role in the treatment of pilon fractures, particularly those with severe metaphyseal comminution and joint involvement. Fibular fixation not only facilitated tibial plafond reduction but also helped maintain the appropriate length and alignment of the tibia.

To minimize soft tissue compromise, we propose the use of retrograde intramedullary K-wires as an alternative to fibular fixation in addition to plating for pilon fracture. As an alternative method for rigid fixation with the plate for the distal fibular fracture at the acute stage, we found that the clinical and radiographic outcomes at postoperative 12 months were also satisfying. We hypothesized that applying locking plates to the tibia would provide sufficient rigidity and stability to the entire ankle joint during nonweight-bearing exercise before callus formation. We noted that none of the patients who underwent fibular intramedullary nailing required further revision surgery involving fibular plating or ankle arthrodesis. Moreover, we determined that earlier surgery involving intramedullary fibular fixation was associated with more favorable functional outcomes. As demonstrated by our findings, a shorter period of hospital admission has the benefit of minimizing socioeconomic burdens.

Numerous studies have revealed the biomechanical and clinical advantages of intramedullary fibular fixation. Systematic reviews of intramedullary fibular fixation for distal fibular fractures have outlined benefits such as a lower risk of complications, faster healing, accelerated rehabilitation, and shorter hospital stays [26,27,28]. In a cadaveric biomechanical study, modern fibular rods demonstrated less external rotation stiffness while maintaining syndesmotic diastasis in AO/OTA 44C^2^ ankle fractures [29]. Considering the lack of research into pilon fractures involving distal fibular fractures, we recommend the execution of additional studies to confirm our hypothesis. Research in this decade has raised concerns about differences in tibial fixation procedures interfering with surgical outcomes for pilon fractures. Considering injury mechanisms, in addition to prioritizing soft tissue conditions, tibial fixation locations can be emphasized to counteract deforming forces [13]. Hong et al. recently conducted a retrospective study and revealed no significant differences in mechanical complications between the different fracture fixation methods [30]. In our study, because of the small sample size, performing further subgroup analysis to investigate the possible relationships between tibial fixation locations and intramedullary fibular fixation was challenging.

In addition to the small sample size, our study has several limitations. First, the study population was selected from only our hospital. The majority of our patients were blue-collar workers, overseas fishers, or farmers. A large proportion of the patients lived in rural areas without easy access to rehabilitation clinics; consequently, tracking medical compliance was difficult. The nature of the patients’ occupations engendered limitations in establishing longer follow-up periods. Furthermore, the patients’ socioeconomic differences may have played a role in selection bias. Although the surgical procedures were performed by qualified orthopedic doctors, bias still existed, especially over the selection of implants. Younger specialists were significantly more likely to favor fibular locking plates. Different types and brands of locking plates may have also interfered with surgical outcomes. Considering the limitations of retrospective studies, we attempted to control for other common confounding factors such as preoperative characteristics (i.e., age, past medical conditions, injury mechanisms, and fracture types). By excluding patients with AO/OTA type III open fractures and those with other severe associated injuries, we could limit the influence of confounders on our results. In addition, demographic data were similar between the groups. According to our review of the literature, this is the first clinical study to explore this topic. Alternative to the stable construct of plate fixation for distal fibula fracture in concomitant of pilon fracture, retrograde intramedullary K-wire pinning for this fracture site may have short-term comparable functional results with the advantage of minimizing surgery-related complications.

## 5. Conclusions

The management of pilon fractures combined with distal fibular fractures is among the major challenges for orthopedic traumatologists. Intramedullary K-wire pinning for distal fibular fractures under specific conditions may achieve comparable short-term postoperative clinical and radiographic alignment outcomes and be associated with a shorter length of hospital stay and less total admission cost with plate fixation for distal fibular fracture in concomitant with plate fixation for pilon fracture. The adequate choice of both fibular fixation methods based on the local soft tissue condition may achieve the same satisfying postoperative OMAS outcomes. In addition, old age, a short time period to surgery, and smoking may be associated with poor OMAS outcomes.

## Figures and Tables

**Table 1:** Demographics of patients with pilon and distal fibula fractures.

Characteristics	Group A (*n* = 80)	Group B (*n* = 76)	Total (*n* = 156)	*p*
Age, mean, ± SD years	54.1 ± 20.5	55.4 ± 15.6	54.7 ± 18.2	0.312
Age group, *n* (%)	-	-	-	0.325
<65 years	60 (75.0%)	58 (76.3%)	118 (75.6%)	
≥65 years	20 (25.0%)	18 (23.7%)	38 (24.4%)	
Sex, *n* (%)	-	-	-	0.548
Male	48 (60.0%)	46 (60.5%)	94 (60.3%)	
Female	32 (40.0%)	30 (39.5%)	62 (39.7%)	
Smoking	9 (11.3%)	8 (10.5%)	17 (10.9%)	0.262
Chronic renal failure	5 (6.3%)	4 (5.3%)	9 (5.8%)	0.210
DM	6 (7.5%)	8 (10.5%)	14 (9.0%)	0.315
Hypertension	24 (30.0%)	20 (26.3%)	44 (28.2%)	0.241

Data are presented as odds ratio (95% CI).

**Table 2:** Injury characteristics and fracture types of pilon fractures and distal fibula fractures.

Characteristics	Group A (*n* = 80)	Group B (*n* = 76)	Total (*n* = 156)	*p*
Injury mechanism, *n* (%)	-	-	-	0.532
High	52 (65.0%)	54 (71.1%)	104 (66.7%)	
Low	28 (35.0%)	22 (28.9%)	50 (33.3%)	
Open facture, *n* (%)	16 (20.0%)	13 (17.1%)	29 (18.6%)	0.351
Distal fibula fracture types, *n* (%)	-	-	-	0.762
Comminuted	26 (32.5%)	28 (36.8%)	54 (34.6%)	
Noncomminuted	54 (67.5%)	48 (63.2%)	102 (65.4%)	
Pilon fracture AO/OTA types, *n* (%)	-	-	-	0.238
Non-C	62 (77.5%)	55 (72.3%)	117 (75.0%)	
Type C	18 (22.5%)	21 (27.7%)	39 (25.0%)	

Data are presented as odds ratio (95% CI).

**Table 3:** The length of hospital stays and the postoperative clinical and radiological outcomes.

	Group A (*n* = 80)	Group B (*n* = 76)	Total (*n* = 156)	*p*
Hospital stays, mean ± SD days	11.4 ± 5.3	18.2 ± 6.8	14.6 ± 4.8	0.024 *
Preoperation, mean ± SD days	4.8 ± 3.3	5.6 ± 3.2	5.0 ± 3.1	0.263
Postoperation, mean ± SD days	6.8 ± 3.2	11.4 ± 4.7	8.7 ± 4.3	0.012 *
Total admission cost (USD)	3624 ± 612	6145± 814	5152 ± 809	0.004 *
Complications, *n* (%)				
Delayed union	11 (13.8%)	14 (18.4%)	25 (16.0%)	0.565
Post-traumatic osteoarthritis	30 (37.5%)	25 (32.9%)	55 (35.2%)	0.613
Wound complications	18 (22.5%)	25 (32.9%)	43 (27.6%)	0.104
Frequency of outpatient visits, *n* (%)	-	-	-	0.697
High	14 (17.5%)	12 (15.8%)	26 (16.7%)	
Low	66 (82.5%)	64 (84.2%)	130 (83.3%)	
LDTA, *n* (%)	-	-	-	0.614
≤10°	68 (85.0%)	66 (86.8%)	134 (85.9%)	
>10°	12 (15.0%)	10 (13.2%)	22 (14.1%)	
ADTA, *n* (%)	-	-	-	0.868
≤10°	66 (82.5%)	63 (82.9%)	129 (82.7%)	
>10°	14 (17.5%)	13 (17.1%)	27 (17.3%)	
OMAS, mean ± SD score	75.4 ± 14.8	77.2 ± 15.6	75.9 ± 15.3	0.523

Data are presented as odds ratio (95% CI); * *p* < 0.05 was considered statistically significant after test; abbreviations: LDTA, lateral distal tibial angle; ADTA, anterior distal tibial angle; OMAS, Olerud and Molander Ankle Score.

**Table 4:** Factors associated with better OMAS among all patients (*n* = 156).

	Crude		Adjusted	
	β (95% CI)	*p*	β (95% CI)	*p*
Age	−0.35 (−0.56, −0.11)	0.003 *	−0.21 (−0.36, −0.02)	0.008 *
Sex (male vs. female)	−1.79 (−6.36, 5.46)	0.612	−1.66 (−7.33, 3.82)	0.542
Fibula fixation method (intramedullary K-pins vs. locking plates)	1.24 (−5.29, 7.76)	0.707	−2.04 (−7.54, 3.46)	0.464
Injury mechanism (high vs. low)	−7.12 (−14.32, 1.93)	0.213		
Fibula fracture type (noncomminuted vs. comminuted)	2.62 (−5.45, 9.67)	0.632		
Time before operation (preoperative admission period)	−0.77 (−1.42, −0.15)	0.005 *	0.83 (0.21, 1.62)	0.018 *
Smoking	−3.23 (−7.73, −0.28)	0.006 *	−2.42(−7.73, −0.28)	0.012 *
Chronic renal failure	−5.69 (−26.32, 15.45)	0.583		
DM	−3.32 (−8.73, 6.31)	0.642	−2.01 (−4.11, 3.23)	0.118
Hypertension	−4.74 (−9.38, 4.01)	0.417		

Data are presented as odds ratio (95% CI); * *p* < 0.05 was considered statistically significant after test; abbreviations: OMAS, Olerud and Molander Ankle Score.

## Data Availability

Data are contained within the article.
